# Development of a model to predict recurrence after bronchial artery embolization for non-cancer related hemoptysis

**DOI:** 10.1186/s12890-021-01790-2

**Published:** 2021-12-18

**Authors:** Hai-Tao Yan, Guang-Dong Lu, Xiang-Zhong Huang, Da-Zhong Zhang, Kun-Yuan Ge, Jin-Xing Zhang, Jin Liu, Sheng Liu, Hai-Bin Shi, Qing-Quan Zu

**Affiliations:** 1 Department of Interventional Radiology, The First Affiliated Hospital With Nanjing Medical University, No. 300 Guangzhou Road, Nanjing, 210029 China; 2 Department of Interventional Radiology, Jiangyin People’s Hospital, Wuxi, 214400 China; 3 Department of Interventional Radiology, Jiangsu Taizhou People’s Hospital, Taizhou, 225300 China; 4 Department of Interventional Radiology, Yixing People’s Hospital, Wuxi, 214200 China; 5 Department of Clinical Medicine Research Institution, The First Affiliated Hospital with Nanjing Medical University, Nanjing, 210029 China

**Keywords:** Bronchial arteries, Embolization, Therapeutic, Hemoptysis, Nomograms, Recurrence

## Abstract

**Background:**

Relapse after effective bronchial arterial embolization (BAE) for controlling hemoptysis is not uncommon. Studies reported diverse predictors of recurrence. However, a model to assess the probability of recurrence in non-cancer related hemoptysis patients after BAE has not been reported. This study was to develop a model to predict recurrence after BAE for non-cancer related hemoptysis.

**Methods:**

The study cohort included 487 patients who underwent BAE for non-cancer-related hemoptysis between January 2015 and December 2019. We derived the model’s variables from univariate and multivariate Cox regression analyses. The model presented as a nomogram scaled by the proportional regression coefficient of each predictor. Model performance was assessed with respect to discrimination and calibration.

**Results:**

One-month and 1-, 2-, 3- and 5-year recurrence-free rates were 94.5%, 88.0%, 81.4%, 76.2% and 73.8%, respectively. Risk factors for recurrence were underlying lung diseases and the presence of systemic arterial-pulmonary circulation shunts. This risk prediction model with two risk factors provided good discrimination (area under curve, 0.69; 95% confidence interval, 0.62–0.76), and lower prediction error (integrated Brier score, 0.143).

**Conclusion:**

The proposed model based on routinely available clinical and imaging features demonstrates good performance for predicting recurrence of non-cancer-related hemoptysis after BAE. The model may assist clinicians in identifying higher-risk patients to improve the long-term efficacy of BAE.

## Introduction

Roughly 70% of hemoptysis is caused by respiratory diseases unrelated to cancer [1–3]. Bronchial arterial embolization (BAE) is highly effective in controlling hemoptysis. However, recurrence of hemoptysis after successful BAE is not uncommon. Extended analysis found that 5–10% of recurrences occurred within one month after BAE primarily secondary to missed culprit arteries [4]. Long-term recurrence rates fluctuated between 20 and 40% in patients with non-cancer-related hemoptysis [3, 5, 6]. Recanalization, revascularization due to migration of embolized materials, and primary diseases progression remained dominant causes of these recurrent events [4, 7, 8]. Approximately 40–60% of patients with recurrent hemoptysis had to undergo repeat embolization, lobectomy, or died [4, 9, 10]. Given these disparate outcomes, an accurate model to predict recurrent hemoptysis would permit more optimal surveillance, prevention, and management strategies for these patients.

Characteristics, including patient demographics, primary respiratory diseases, thoracic imaging, and embolization procedures, were analyzed for significant prediction values of recurrence [3, 4, 9–13]. BAE showed its superiority of controlling emergency bleeding by embolizing target vessels, not eliminate underlying lung diseases with equivalence effect of curative intent, e.g., lobectomy. Pre-procedure CT imaging and angiographic findings were also assessed for their predictive capability [4]. However, those studies did not fully integrate the clinical and imaging features nor determine their individual contributions to predicting recurrent bleeding. In addition, some researchers combined non-cancer and cancer related hemoptysis in the analysis [14]. In light of the discrepant nature of benign and malignant diseases-associated hemoptysis, a prediction model specific to each group is needed.

In this study, we employed a large, multi-center cohort to develop a risk prediction model for recurrence of non-cancer related hemoptysis after endovascular treatment. The model can be used in evaluating individualized prognosis and in raising precaution for the risk cohort.

## Patients and methods

Ethics committee approval was granted by the local institutional ethics review board for this retrospective study. The requirement for informed consent was waived due to its retrospective nature. All procedures followed in this study conformed to the guidelines of the World Medical Association’s Helsinki Declaration (2008). The data that support the findings of this study are available from the corresponding author upon reasonable request.

### Patients

**Fig. 1:**
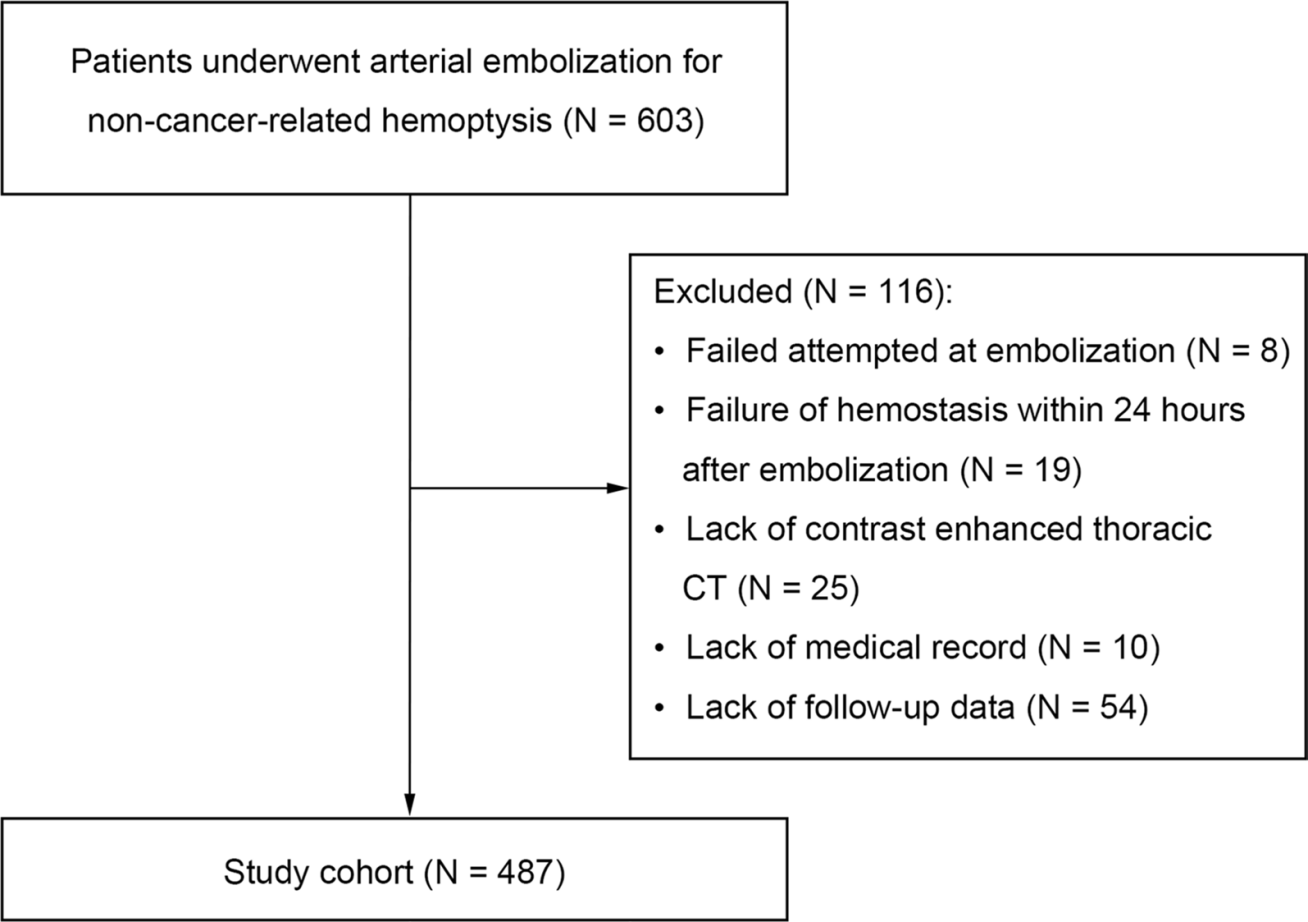
Flowchart of enrolled patients

The medical records of 603 patients who underwent arterial embolization for non-cancer-related hemoptysis at four tertiary centers between January 2015 and December 2019 were queried. During hospitalization, all hemoptysis patients underwent the standard care: vital sign monitoring, keeping airway stabilization, correction of hypoxemia, hemostasis, nebulized treatment and empirical antibiotic therapy. The exclusion criteria were: (1) either technique failure (failed attempted at embolization) or clinical failure (failure of hemostasis within 24 h after embolization) [15]; (2) lack of contrast enhanced thoracic computed tomography (CT); (3) medical record missing; and (4) lack of follow-up data. Based on the criteria, 487 patients (348 men and 139 women) were included in the study cohort (Fig. 1). From the medical records, information deemed related to hemoptysis recurrence, including patient demographics, clinical features, radiographic findings, and embolization procedure, were gathered. A subgroup of the study cohort, patients were diagnosed as bronchiectasis (n = 251), was analyzed and published in the Journal of CardioVascular and Interventional Radiology.

Hemoptysis severity were graded according to blood loss within 24 h as mild (< 100 ml), moderate (100–300 ml), and massive (≥ 300 ml) [2]. The CT angiography (CTA) was performed in our study with SOMATOM Force, Siemens, Germany; Revolution CT, GE Healthcare, United States; and Optima CT, GE Healthcare, United States with approximately 50–60 mL of contrast agent (Iodixanol 320 mgI/mL, GE Healthcare or Iopromide 370 mgI/mL, BAYER), which was injected intravenously at a speed of 4.0 mL/s. Then all image data were transferred to a post-processing workstation and reconstructed at 1.25 mm section thickness. The culprit vessels on CTA were identified upon hypertrophy, hypervascularity, tortuosity, and aneurysmal. Patients were classified into three categories disease categories: bronchiectasis, tuberculosis (TB) sequela, and non-TB and non-bronchiectasis (chronic pneumonia, cryptogenic hemoptysis, aspergilloma and pneumoconiosis). Cryptogenic hemoptysis indicated the lack of specific lung parenchymal or vascular abnormality as noted on preoperative CTA [16]. The extent of pulmonary disease (number of affected lobes), presence of pleural thickening, and lung destruction were assessed. Pleura thickness > 3 mm was considered pathological [17]. Lung destruction was defined as diffuse lung parenchymal destruction or lung volume loss in at least one lobe on CT [18].

### Arterial embolization procedures

Before procedure, interventional radiologists reviewed CTA to observe the lung lesion, culprit artery anatomy. We also searched suspicious non-bronchial systemic arteries (NBSAs) according to the location of lung lesion, including inferior phrenic arteries for lower lung lesion, internal mammary or esophageal arteries for medial lung lesion and superior thoracic arteries for upper lung lesion, etc. BAE was performed with Artis Zeego Digital Subtraction Angiography (DSA), Siemens, Germany; IGS5-4 DSA, GE, United States; UNIQ FD20 DSA, PHILIPS, Netherlands and UNIQ FD20/15 DSA, PHILIPS, Netherlands. All procedure was done via a transfemoral approach under local anesthesia. Angiography was performed by a 5F catheter (Cobra, RLG, MIK catheter, Cook Medical, Bloomington, Indiana, USA) with a total amount of 6-10 mL contrast agent at a rate of 1.5–2.0 mL/s. Selective angiography of the bronchial arteries or suspicious NBSAs were recorded. Abnormal angiographic findings included abnormal culprit artery hypertrophy, distortion, neovascularity, hypervascularity, systemic arterial-pulmonary circulation shunts (SPS) or extravasation of the contrast agent [19]. Once the presence of abnormal vessels was confirmed, a microcatheter (2.7F; Terumo Medical Corp., Tokyo, Japan; or 2.4F; Merit Maestro, South Jordan, Utah, USA) was introduced and advanced to avoid the possible risk of non-target embolization. Embolization continued until complete occlusion of all bronchial and pathological NBSA was achieved. Embolization materials included polyvinyl alcohol (PVA) particles (300–500 μm; Cook), microspheres (500–700 μm; Merit Maestro), and gelatin sponge particles (350–560 μm; Hangzhou Alicon Pharmaceutical Co., Ltd., Zhejiang, China). Micro-coils (COOK) were used in 25 patients after initial embolization with particles. Procedure-related complications were classified according to the Society of Interventional Radiology (SIR) guidelines [20].

### Follow-up and recurrence

After discharge, patients were followed up by outpatient clinic during 1–3 months and then underwent telephone interview for the first two years. If no recurrence, follow-up cycle was extended to once a year. During the follow-up, healthy lifestyle education, antibiotics treatment and rehabilitation was encouraged for the better long-term outcome. Recurrence was defined as hemoptysis ≥ 30 ml per day and needing further medical care, repeat BAE, lobectomy, or hemoptysis as a cause of death after clinical success [11, 21]. The end date of follow-up was defined as date of death by any cause or May 2020. Recurrence-free time was the period from the date of embolization procedure to the date of recurrence, death, or last follow-up.

### Development and validation of the prognostic model

We performed univariate and multivariate Cox regression analyses to determine recurrence risk factors from clinical and imaging signatures. Variables with significance in the univariable Cox regression analysis were included in the multivariate Cox analysis with backward selection method. The candidate clinical variables were age, sex, underlying lung disease, amount of hemoptysis, history of hemoptysis, smoking, hypertension, and previous lobectomy. Imaging features included the extent of pulmonary disease (number of affected lobes), presence of pleural thickening, lung destruction, number of culprit bronchial arteries, SPS, presence of culprit NBSAs, and type of embolization material.

Based on the results of multivariate regression, the risk prediction model for recurrence was formulated and presented with a nomogram. The regression coefficient of variables derived from multivariate Cox regression analysis was proportionally converted to a 0- to 100-point scale and each variable was listed separately. The points of the independent variables were added to derive total points, which was matched to a scale of outcome. The performance of the model was measured by the area under the receiver operating characteristic (ROC) curve (AUC) value, calibration curves, and Brier score.

### Statistical analysis

Baseline data were expressed as mean (SD) or median (range) for quantitative variables and frequencies (percentage) for categorical variables. We used Kaplan–Meier analysis to estimate cumulative recurrence-free curves. Univariate and multivariate Cox regression analyses were applied to identify the independent predictors of recurrent hemoptysis. Bootstrapping with 1000 samples was used for model calibration validation. The discrimination performance of our model was quantified by the AUC value. The “Boot632plus” split method with 1000 iterations was used to calculate the prediction error of the current model. Estimates of prediction error were summarized as the integrated Brier score, which meant a weighted average of the squared distances between predicted probabilities and observed outcomes. The Brier score can reflect discrimination and calibration simultaneously; it can evaluate the overall model performance effectively. When a model is perfect, the Brier score will approach 0.0. For a model with a 50% incidence of the outcome, the Brier score will equal 0.25 [22]. All statistical analyses were conducted with software (R, version 4.0.2). *P*-values below 0.05 were considered statistically significant.

## Results

### Characteristics of the study cohorts

Table 1 shows baseline characteristics of the study patients. Median duration of hemoptysis was 7 days (range, 0 day–35 years). The common underlying lung diseases were bronchiectasis (251/487, 51.5%) and TB sequela (139/487, 28.5%). In total, 1279 arteries including 1047 bronchial arteries (569 in right, 478 in left) and 232 NBSAs, were embolized.

**Table 1:** Baseline characteristics of the study patients (N = 487)

Variables	Mean ± SD/n (%)
Age (years)	60.2 ± 13.5
Sex (female/male)	139 (28.5%)/348 (71.5%)
Median duration of hemoptysis (range, d)	6 (0–12,775)
Hemoptysis amount (ml/d)	
< 100	189 (38.8%)
100–300	212 (43.5%)
≥ 300	86 (17.7%)
Underlying lung disease	
Bronchiectasis	251 (51.5%)
TB sequela	139 (28.5%)
Non-TB and non-bronchiectasis^#^	97 (19.9%)
History of lobectomy	12 (2.5%)
Smoking	145 (29.8%)
Hypertension	141 (29.0%)
Disease extent (number of affected lobes)	2.4 ± 1.0
Presence of pleural thickening	270 (55.4%)
Lung destruction	29 (6.0%)
Number of culprit bronchial arteries	2.2 ± 1.0
Presence of culprit NBSAs	134 (27.5%)
Systemic arterial-pulmonary circulation shunts	173 (35.5%)
Embolization materials	
PVA particle	394 (80.9%)
Microsphere	70 (14.4%)
Gelatin sponge	23 (4.7%)
Median follow-up duration (range, d)	675 (143–1943)
Recurrence	93 (19.1%)

NBSAs, non-bronchial systemic arteries; PVA, polyvinyl alcohol; SD, standard deviation; TB, tuberculosis

^#^Non-TB and non-bronchiectasis included chronic pneumonia (n = 55), cryptogenic hemoptysis (n = 32), aspergilloma (n = 1) and pneumoconiosis (n = 9)

### Complication and recurrence

Minor complications occurred in 80 patients, including fever in 36, chest or shoulder discomfort in 30, abdominal discomfort or vomiting in 10, puncture site hematoma in two, headache in one, and anaphylaxis in one patient. One patient experienced a major complication. He suffered left extremity weakness one day after procedure and CT images showed cerebral lacunar infarction. This patient presented with bronchial artery-pulmonary artery shunt and was embolized with 300–500 μm PVA particles. The modified Rankin Scores were 2 at discharge and remained stable at the three years’ follow-up visit.

The median follow-up time was 675 days (range, 143–1943 days). Recurrent hemoptysis events occurred in 93 (19.10%) cases. Eighty (86.0%) of the recurrent cases occurred within 2 years after BAE. The one-month, and 1-, 2-, 3- and 5-year cumulative recurrence-free rates of patients were 94.5%, 88.0%, 81.4%, 76.2% and 73.8%, respectively. Among all patients with recurrence events, 47 (50.5%) patients underwent repeated BAE, one (1.1%) underwent a lobectomy, seven (7.5%) died due to recurrence and 38 (40.9%) received conservative management. Repeat BAE showed that causes of recurrent hemoptysis included missed culprit arteries (n = 11, 23.4%), recanalization (n = 20, 42.6%) and revascularization of the collateral circulation (n = 16, 34.0%).

### Development and validation of the prognostic model

**Fig. 2:**
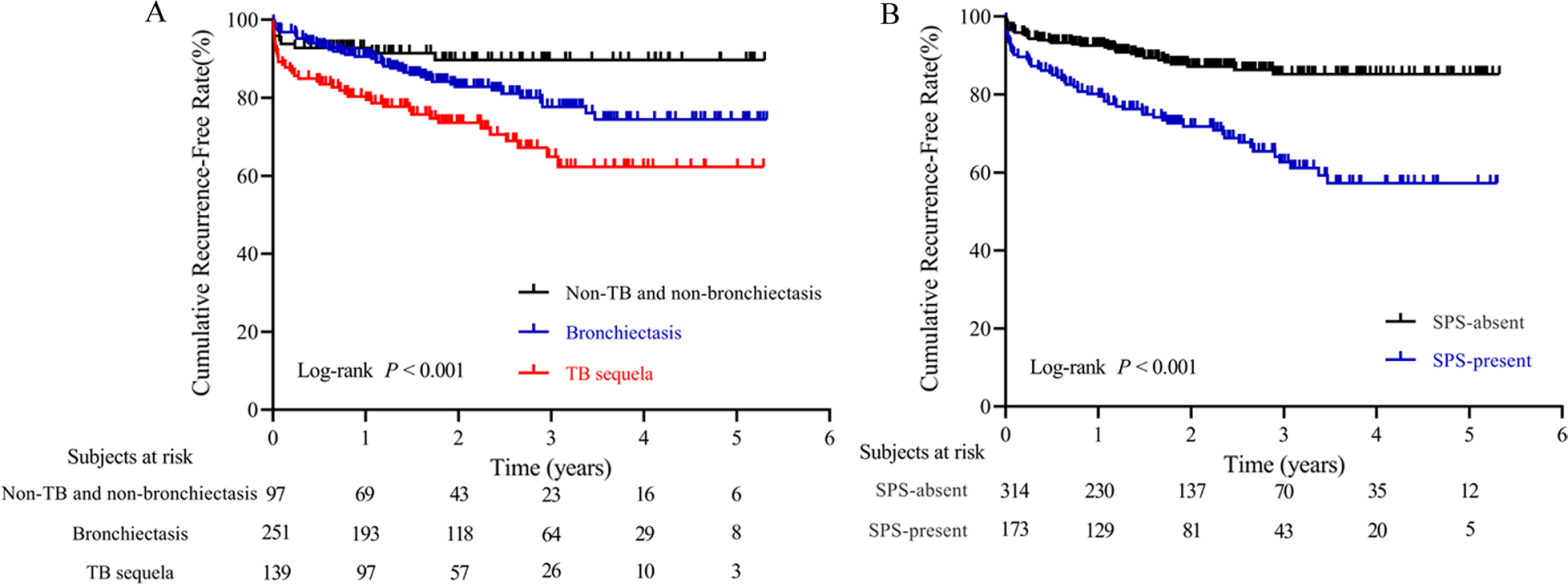
Kaplan–Meier curves for recurrence-free time in all patients based on underlying lung diseases (**A**) and presence of systemic arterial-pulmonary circulation shunts (**B**)

**Fig. 3:**
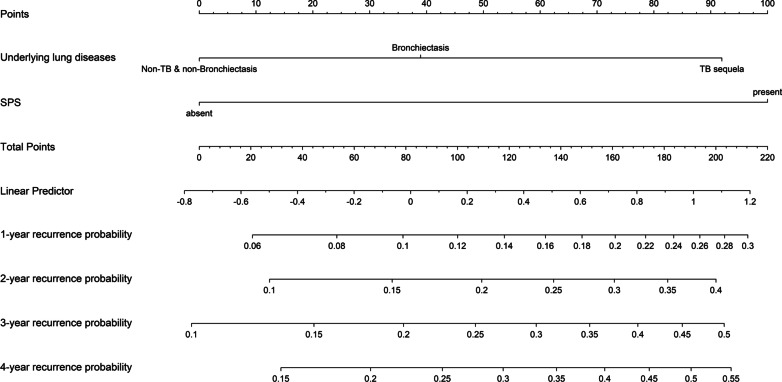
The prognostic model presented with a nomogram scaled by the proportional regression coefficient of each predictor: underlying lung diseases and systemic arterial-pulmonary circulation shunts

**Fig. 4:**
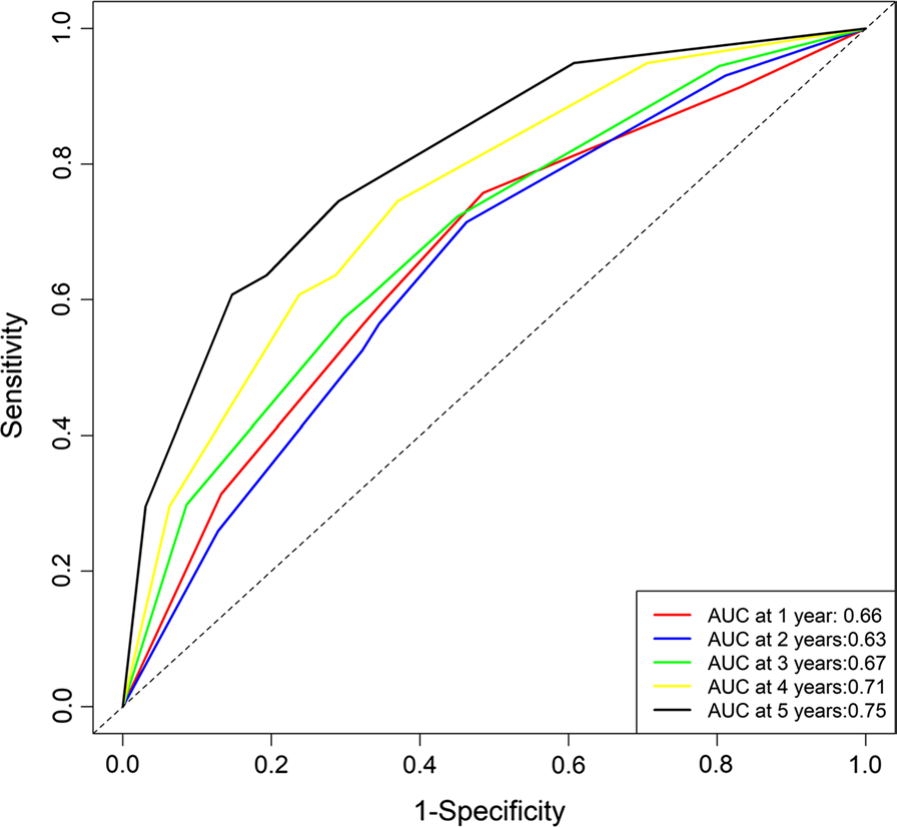
The time-dependent ROC curve analysis for nomogram for patient cohorts

**Fig. 5:**
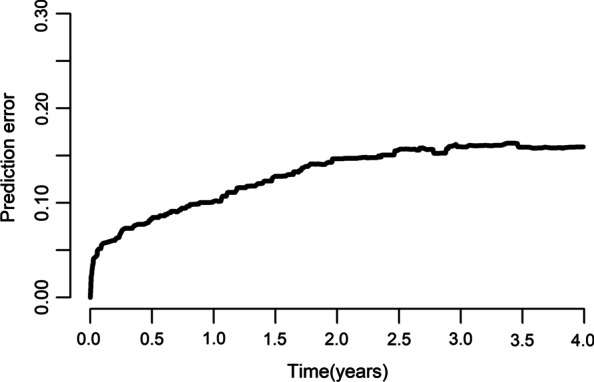
The time-dependent prediction error rate for the model

**Fig. 6:**
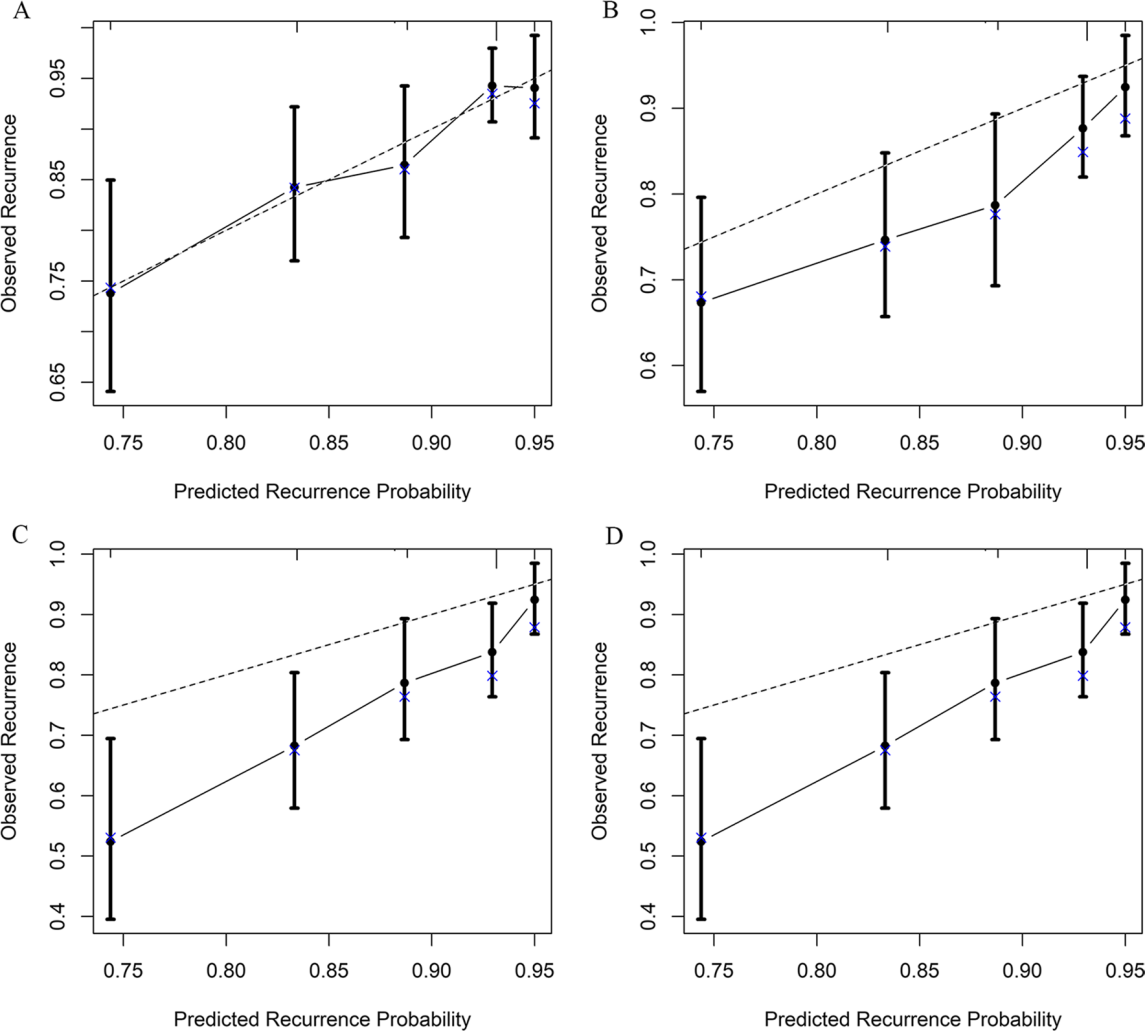
**A**–**D** The time-dependent calibration curve of the model at 1-, 2-, 3-, and 4-year after BAE

Univariate analysis showed that sex, underlying lung diseases, history of hemoptysis, history of lobectomy, presence of pleural thickening, lung destruction, the extent of pulmonary disease (number of affected lobes), SPS, presence of culprit NBSAs and type of embolization material were statistically significant factors affecting recurrence. Multivariate analysis revealed that underlying lung diseases (P = 0.026) and SPS (P < 0.001) were most significant of recurrence (Table 2). Recurrence-free curves for patients classified by underlying lung diseases and the presence of SPS are shown in Fig. 2. Based on these findings, a nomogram for individual hemoptysis patient risk stratification was created (Fig. 3). The 1-year, 2-year, 3-year and 4-year recurrence rates of individual patients could then be predicted after embolization. The model maintained good discriminatory performance (AUC, 0.69; 95% CI 0.62–0.76). The time-dependent AUC value is shown in Fig. 4. The prediction error of our model was low (integrated Brier score, 0.143). The prediction error rate of recurrence at 4 years was about 20% (Fig. 5). The calibration chart between predicted and observed recurrence probability was plotted. The accuracy of prediction for first 2-year recurrence was acceptable, while late recurrence prediction ability declined moderately (Fig. 6A–D).

**Table 2:** Univariate and multivariate analysis of the variables associated with recurrence of hemoptysis in patients after BAE treatment

Variables	Level	N	Univariate		Multivariate	
			HR (95% CI)	*P* value	HR (95% CI)	*P* value
Age	–	487	1.01 (0.99–1.03)	0.299		
Sex				0.048		
	Male	348	0.65 (0.43–1.00)			
	Female	139	Reference			
The history of hemoptysis				< 0.001		
	> 6 months	163	2.47 (1.64–3.71)			
	≤ 6 months	324	Reference			
Hemoptysis amount				0.771		
	≥ 300 ml/d	86	1.21 (0.68–2.16)	0.508		
	100–300 ml/d	212	1.14 (0.72–1.79)	0.583		
	< 100 ml/d	189	Reference			
Underlying lung diseases				0.001		0.026
	TB sequela	139	3.32 (1.61–6.85)	0.001	2.32 (1.15–5.19)	0.027
	Bronchiectasis	251	1.84 (0.90–3.77)	0.096	1.43 (0.72–3.16)	0.338
	Non-TB and non-bronchiectasis	97	Reference		Reference	
History of lobectomy				0.015		
	Present	12	3.07 (1.24–7.60)			
	Absent	475	Reference			
Smoking				0.298		
	Present	145	0.78 (0.49–1.25)			
	Absent	342	Reference			
Hypertension				0.240		
	Present	141	0.75 (0.47–1.21)			
	Absent	346	Reference			
Disease extent (number of affected lobes)	–	487	1.42 (1.18–1.72)	< 0.001		
Presence of pleural thickening				< 0.001		
	Present	270	2.39 (1.52–3.76)			
	Absent	217	Reference			
Lung destruction				< 0.001		
	Present	29	4.20 (2.44–7.20)			
	Absent	458	Reference			
Number of culprit BAs	–	487	0.87 (0.70–1.08)	0.212		
Presence of culprit NBSAs				0.004		
	Present	134	1.84 (1.21–2.78)			
	Absent	353	Reference			
Systemic arterial-pulmonary circulation shunts				< 0.001		< 0.001
	Present	173	2.86 (1.88–4.34)		2.49 (1.63–3.87)	
	Absent	314	Reference		Reference	
Embolization materials				0.004		
	Gelatin sponge	23	2.74 (1.38–5.46)			
	PVA particle and microsphere	464	Reference			

BA, bronchial arteries; BAE, bronchial arterial embolization; CI, confidence interval; HR, hazard ratio; NBSAs, non-bronchial systemic arteries; PVA, polyvinyl alcohol; TB, tuberculosis; SD, standard deviation

## Discussion

Results from our study showed BAE was highly effective in controlling hemoptysis, with 2-year and 5-year recurrence-free rates of 81.4% and 73.8%, respectively. However, the cumulative recurrent rates did not improve significantly over a decade [2]. Based on the clinical and imaging parameters in this multi-institutional study, the prediction model accounted for the interaction of relevant and statistically significant factors to predict the recurrence of non-cancer-related hemoptysis in patients after embolization. Two factors, underlying lung diseases and SPS, were found most useful at predicting recurrence and incorporated into the model. The objectiveness and accessibility properties of these factors suggest that clinical application of the model will be straightforward. Results showed the model possessed good prognostic ability (AUC, 0.69; 95% CI 0.62–0.76) and a lower prediction error (integrated Brier score, 0.143).

We enrolled non-cancer related hemoptysis patients with technical and clinical successful embolization procedures in this study to develop the model. The 1-, 2-, 3- and 5-year cumulative recurrence-free rates were 88.0%, 81.4%, 76.2% and 73.8%. This was a promising long recurrence-free interval and could be explained by the differences in enrolled population, adequate preoperative CTA evaluation, reasonable antibiotic therapy and relative regular follow-up. During the median follow-up time of 675 days, 80 patients (80/93, 86.0%) experienced relapse within 2 years, while few recurrences occurred later. This 2-year recurrent peak period was also noted by others [9, 10]. Taking this into consideration, patients should be closely followed-up for at least 2 years after the embolization. Calibration performance showed the model had a higher predictive power in the first 2 years' events. This inadequacy of the model to predict recurrence beyond 2 years after BAE may be explained by the nature of the underlying lung diseases. TB sequela and bronchiectasis with chronic inflammatory features accounted for 28.5% and 51.5% of our study population, respectively. During this 2-year period, arteriogenesis was likely driven by inflammatory mediators [23]. While embolization initially achieved stasis, promoting recovery and stabilization following embolization, long-term outcome of individuals of those respiratory inflammatory disorders would inevitably be affected by unquantifiable factors, such as climate, daily habits and comorbidities.

Pre-procedure CT and culprit vessels embolization reduced the post-BAE relapse [24]. After successfully controlling hemoptysis, our model showed the accurate prediction of the probability of relapse was independent of the underlying lung diseases. In chronic pathophysiological condition, proangiogenic factors, such as vascular endothelial growth factor and angiopoietin-1, were secreted, which promoted the development of new fragile bronchial vasculature and the remodeling of existing vessels [25, 26]. Consistent with previous studies [9, 12, 13], TB sequela was more commonly associated with recurrence. These findings argue for aggressive management of primary lung diseases to reduce hemoptysis recurrence. Therefore, we constructed a subgroup analysis focusing on idiopathic bronchiectasis to remove the influence of the underlying lung diseases and provide the corresponding treatment for this specific benign disease. And the results showed that the nomogram based on the idiopathic bronchiectasis patients maintained favorable predictability for recurrence and improved long-term outcomes of patients at risk of recurrence are expected with thorough follow-up, health education, and pulmonary rehabilitation [27]. As previous reported, SPS, as a specific angiographic finding during procedure, was again demonstrated to be an independent risk factor for recurrence after BAE [3, 4, 10, 14]. These two factors encompassed clinical and imaging parameters, and as part of our model, resulted in good discriminatory prediction and applicability. However, the existence of SPS and its impact on recurrence is not uniformly acknowledged [4, 9, 10, 28]. Normally, the systemic and pulmonary circulations communicate at the capillary and precapillary levels [29]. If the pulmonary circulation was compromised, impairment of the pulmonary circulation and an increased demand for oxygen in local lung tissue happened. Systemic arteries supplying the lung, mainly bronchial arteries, often increase their flow to compensate for decreased lung perfusion [30, 31]. However, under such scenarios, the communications between the systemic arteries and pulmonary micro-circulation are prone to bleeding [32]. In general, BAE in individuals with SPS was safe. However, one patient with a shunt suffered left extremity weakness one day after procedure and CT images showed multi-lacunar infarction. We inferred that it might be related with BAE procedure, but not sure. The safety and influence of shunts during BAE warrants further study.

Certain limitations of this study should be pointed out. First, the study was retrospective and is predicted to result in bias. To minimize the bias of post-hoc analysis, an internal validation was performed. Ultimately, prospective validation is needed to verify the predictive performance of this model. Second, the primary disease spectrum in our cohort differed from that in previous studies. For example, aspergilloma was reported to be associated with hemoptysis recurrence [9, 33]. However, we could not analyze this risk factor because only one patient in our study was diagnosed with aspergilloma and did not experience relapse. Third, laboratory results were not analyzed, due to their high variability in the context of long-term events prediction. Fourth, the model did not assess the effects of medical treatment for pulmonary diseases that were coincident with or initiated after BAE.

In conclusion, a model incorporating underlying lung diseases and SPS demonstrated good performance for predicting the recurrence of non-cancer-related hemoptysis after embolization. The model may assist clinicians in identifying higher-risk patients to improve the long-term efficacy of BAE.

## Data Availability

The data that support the findings of this study are available from the corresponding author upon reasonable request.

## Abbreviations

BAE: Bronchial arterial embolization
CI: Confidence interval
CTA: CT angiography
DSA: Digital Subtraction Angiography
HR: Hazard ratio
NBSAs: Non-bronchial systemic arteries
PVA: Polyvinyl alcohol
ROC: Receiver operating characteristic curve
SIR: Society of interventional radiology
SPS: Systemic arterial-pulmonary circulation shunts
TB: Tuberculosis
